# FDA-approved Abl/EGFR/PDGFR kinase inhibitors show potent efficacy against pandemic and seasonal influenza A virus infections of human lung explants

**DOI:** 10.1016/j.isci.2023.106309

**Published:** 2023-02-28

**Authors:** Robert Meineke, Sonja Stelz, Maximilian Busch, Christopher Werlein, Mark Kühnel, Danny Jonigk, Guus F. Rimmelzwaan, Husni Elbahesh

**Affiliations:** 1 Research Center for Emerging Infections and Zoonoses (RIZ), University of Veterinary Medicine in Hannover (TiHo), Bünteweg 17, 30559 Hannover, Germany; 2 Department of Pathology, Hannover Medical School (MHH), Carl-Neuberg-Straße 1, 30625 Hannover, Germany; 3 Biomedical Research in End-stage and Obstructive Lung Disease Hannover (BREATH), Member of the German Center for Lung Research (DZL), Hannover Medical School (MHH), Carl-Neuberg-Straße 1, 30625 Hannover, Germany

**Keywords:** Virology, Cell biology

## Abstract

Influenza viruses (IVs) cause substantial global morbidity and mortality. Given the limited range of licensed antiviral drugs and their reduced efficacy due to resistance mutations, repurposing FDA-approved kinase inhibitors as fast-tracked host-targeted antivirals is an attractive strategy. We identified six FDA-approved non-receptor tyrosine kinase-inhibitors (NRTKIs) as potent inhibitors of viral replication of pandemic and seasonal IVs *in vitro*. We validated their efficacy in a biologically and clinically relevant *ex vivo* model of human precision-cut lung slices. We identified steps of the virus infection cycle affected by these inhibitors and assessed their effect(s) on host responses. Their overlapping targets suggest crosstalk between Abl, EGFR, and PDGFR pathways during IAV infection. Our data and established safety profiles of these NRTKIs provide compelling evidence for further clinical investigations and repurposing as host-targeted influenza antivirals. Moreover, these NRTKIs have broad-spectrum antiviral potential given that their kinase/pathway targets are critical for the replication of many viruses.

## Highlights

• Five of six NRTKIs that inhibited IAV replication *in vitro* were validated *ex vivo*
• These NRTKIs inhibited the replication of the H3N2 and H1N1 IAV subtypes
• NRTKIs targeting EGFR, PDGFRα, and Abl displayed the most potent IAV inhibition
• Tested NRTKIs showed a high genetic barrier to the emergence of resistance mutations

## Graphical abstract

**Figure:**
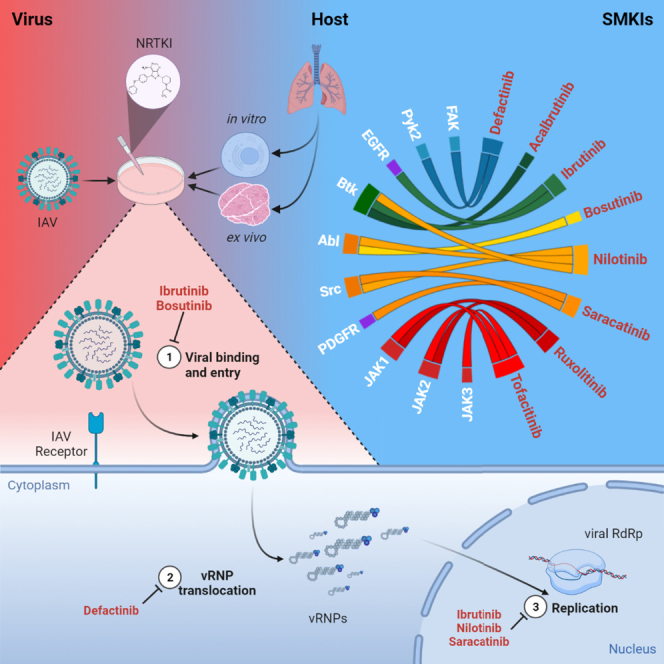

## Introduction

Influenza viruses (IVs) cause respiratory tract infections in humans and are responsible for substantial annual morbidity and mortality, especially in individuals at high risk, like young children, the elderly, or immunocompromised patients. The most important preventative measure of protection from IV infections is vaccination. However, accumulation of genomic mutations contributes to reduced vaccine efficacy and evasion of virus-neutralizing antibodies; necessitating annual update vaccines against seasonal IVs.^1^^,^^2^^,^^3^^,^^4^ Moreover, genetic reassortment that leads to the emergence of novel IVs in human populations, that largely lack virus-neutralizing antibodies, can result in pandemic outbreaks. As with previous influenza pandemics and the current SARS-CoV-2 pandemic, effective vaccines are not readily available at early stages of a pandemic.

In absence of efficacious vaccines, virus-targeted antivirals offer some protection if administered within the therapeutic window. Until recently, only two classes of influenza antivirals were available, adamantanes targeting the viral M2 ion channel protein and neuraminidase inhibitors (NAI). However, adamantanes are ineffective due to resistance mutations in currently circulating strains and are no longer clinically used.^5^ In contrast, only ∼4–5% of currently circulating viruses carry resistance mutations to NAIs (like oseltamivir).^6^ Favipiravir (T705), baloxavir and pimodivir are recently developed antivirals targeting the viral PA, PB1, and PB2 polymerase proteins, respectively, and can inhibit adamantane- and NAI-resistant viruses.^7^^,^^8^ Although baloxavir was approved for the treatment of acute “uncomplicated” influenza infections in the United States in 2018, recent studies have already observed ∼10% of isolates from otherwise healthy adults and adolescents carried baloxavir resistance mutations; this may potentially be higher in immunocompromised patients.^9^^,^^10^^,^^11^^,^^12^

Sustained circulation of virus variants resistant to current antivirals and low genetic barrier for resistance highlight the need for host-targeted therapeutics; that do not suffer from these limitations. Because all viruses rely on cellular machinery for their replication, several host proteins are known to be required for efficient viral replication and pathogenesis.^13^^,^^14^^,^^15^^,^^16^^,^^17^ Host kinases regulate signaling pathways that are critical for replication of many viruses including influenza by direct phosphorylation of viral proteins or their cellular interaction partners.^13^^,^^14^^,^^18^^,^^19^^,^^20^ Although the human kinome consists of >550 kinases, only 62 small-molecule kinase inhibitors (SMKIs) are FDA-approved, primarily used to treat cancers and mostly target tyrosine kinases.^21^ Non-receptor tyrosine kinases (NRTKs) are cytoplasmic/membrane-anchored kinases that closely associate with cellular receptors or receptor complexes to mediate outside-in signaling.^21^^,^^22^^,^^23^^,^^24^^,^^25^^,^^26^ NRTKs like most kinases contain a catalytic kinase domain, and several protein-protein interaction motifs (e.g., SH2, SH3, and PH domains) necessary to relay cell signals. NRTKs including Abl, FAK, JAK, Src, and BTK have all been reported to play a role in IAV infections. Previous studies demonstrated that NRTK inhibitors (NRTKIs) modulate pro- and anti-viral signaling *in vitro* resulting in reduced viral pathogenesis and increased survival *in vivo*.^13^^,^^16^^,^^27^^,^^28^^,^^29^^,^^30^^,^^31^^,^^32^ However, no NRTKIs or SMKIs are approved for clinical use against IVs. Here, we identified, validated, and characterized five currently available FDA-approved NRTKIs as a potent inhibitor of IAV replication *in vitro* and *ex vivo* human lung tissue.

## Results

### NTRKI treatment inhibits IAV replication *in vitro*

**Figure 1:**
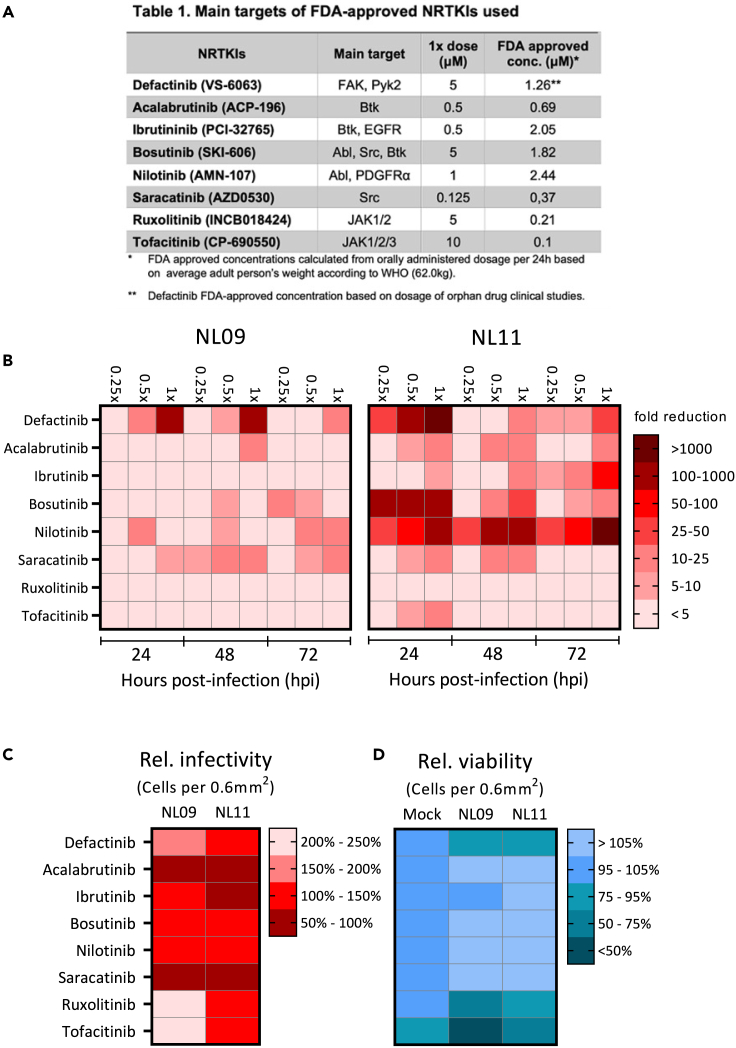
Effect of NRTKI treatment on IAV replication, infectivity, and viability (A) Table 1. Main targets of FDA-approved NRTKIs used in this study. (B) A549 cells were infected with NL09 or NL11 at MOI = 1 +/− indicated NRTKIs at [0.25x, 0.5x, or 1x]_max_ concentration for 72 h. Viral titers were quantified by TCID_50_/mL assay at 24, 48, and 72 hpi and visualized by Heatmap of fold-change in viral titers relative to DMSO treated (n = 4/condition). *Also see*Figure S1. (C and D) A549 cells were infected with NL09 or NL11 at MOI = 1 and incubated for 48 h in the presence of SMKIs ([0.5x]_max_ concentration). Fluorescence microscopy images were acquired from cells stained for infection by anti-IAV NP antibody (red), and nuclei by using NucBlue Live ReadyProbes (blue). Data visualized by Heatmap are % infectivity (C) and % cell viability (D) relative to untreated infected cells or mock-infected treated cells, respectively (n = 4/condition) *Also see*Figure S2. Images quantified by ImageJ software. p values were determined by Mann-Whitney tests compared to untreated cells.

We first identified non-toxic SMKI concentrations (≥90% relative to DMSO-treated cells) using CellTiter-Glo (CTG), an ATP-based cell-viability assay. The highest concentration with ≥90% relative viability is defined as 1x concentration ([1x]_max_) (Figure 1A/Table 1). Next, A549 cells were infected with either pandemic A(H1N1)pdm09 A/Netherlands/602/09 (NL09) or seasonal A(H3N2) A/Netherlands/241/11 (NL11) strains at a multiplicity of infection (MOI) of 1 in presence or absence of [1x, 0.5x and 0.25x]_max_ of respective NRTKIs following inoculation. We observed dose-dependent viral titer reductions (from 2- to 1,000-fold) by six of eight NRTKIs (Figures 1B and S1). We observed variability in the magnitude and duration of titer reductions with more pronounced effects in NL11 (H3N2) infected cells than in NL09 (H1N1) infected cells. Although we observed only a transient reduction at 24 hpi with pan JAK (JAK1/⅔) inhibitor Tofacitinib (TF), we did not observe any significant reduction with the JAK1/2 selective inhibitor Ruxolitinib (RX). The highest and most sustained level of reduction (≥1,000-fold or 3-log_10_) was observed with Nilotinib (NI) (Abl/PDGFRa inhibitor). Bosutinib (BO) (Abl/Src/Btk inhibitor and Saracatinib (SA) (Src inhibitor) also showed inhibition (SA ∼5 to 25-fold; BO ∼10 to 1,000-fold). Acalabrutinib (AC) (Btk inhibitor) had minimal effect on NL09 replication but greater and longer inhibition (5- to 25-fold) of NL11 replication at higher concentrations (0.25 and 0.5 μM). Ibrutinib (IB) (Btk/EGFR inhibitor) had minimal effects on NL09 replication but a more appreciable and sustained (5- to 100-fold) reduction of NL11 replication at the highest concentration used (0.5 μM). Defactinib (DF) (FAK/Pyk2 inhibitor) treatment resulted in robust viral titer reduction (10- to 1,000-fold) in both NL09 and NL11 at higher concentrations (2.5 and 5.0 μM). In DF-treated NL09 infected cells, a reduction was more robust at earlier times (24 and 48 hpi), but in NL11 infected cells a larger reduction was observed at 24 and 72 hpi.

### NRTKIs affect IAV infectivity and cell viability during IAV infection

Next, we determined the effect of NRTKIs on viral infectivity (number of infected cells) and cellular viability (total number of cells) in infected A549 cells by quantitative immunofluorescence microscopy. Treatment with most inhibitors caused a significant increase in infectivity despite reduction of viral titers. We observed robust significant increases in relative infectivity in cells treated with TF (NL09 = 217%, NL11 = 112%), RX (NL09 = 203%, NL11 = 116%), and DF (NL09 = 180%, NL11 = 139%). Among BO- and NI-treated cells, we only observed marginal increases in relative infectivity (105%) in cells treated with BO or NI in NL09-infected cells. We observed marked decreases in relative infectivity in cells treated with AC (NL09 = 78%, NL11 = 74%) and SA (NL09 = 90%). We observed opposite effects on NL09-infected cells following treatment with IB (NL09 = 116%) (Figures 1C and S2).

Next, we determined whether NRTKI treatment improved cell viability during IAV infection. In mock-infected A549 cells treated with tested NRTKIs at [0.5x]_max_, <5% reduction in cell viability was observed (Figure 1D). However, IAV infection caused significant synergistic cytotoxicity and decreased viability with TF (NL09 = 40%, NL11 = 63%), RX (NL09 = 66%, NL11 = 89%), or DF (NL09 = 84%, NL11 = 92%) treatment. However, cell viability was increased by treatment of IAV-infected cells with AC (NL09 = 112%, NL11 = 109%), IB (NL09 = 102%, NL11 = 109%) BO (NL09 = 111%, NL11 = 114%), NI (NL09 = 110%, NL11 = 110%) or SA (NL09 = 150%, NL11 = 127%) (Figure 1D). Notably, this effect was only statistically significant for the treatment with SA. Therefore, neither decreased infectivity nor cell viability alone does not account for the observed NRTKI-induced reduction in viral titers.

### The antiviral effect of NRTKIs is MOI-independent

**Figure 2:**
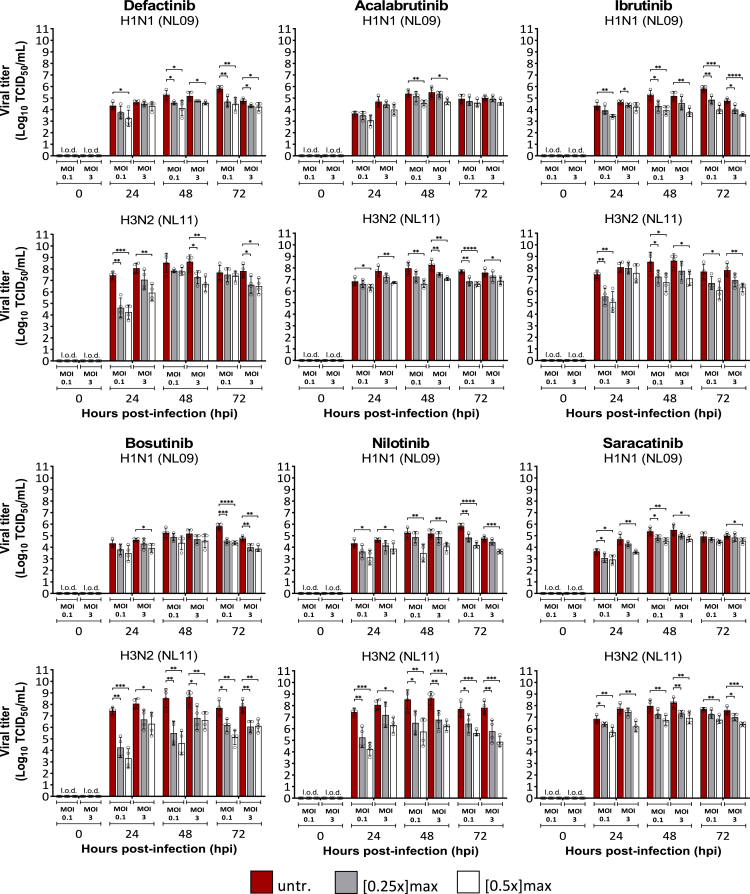
MOI-independent effect of NRTKIs on IAV infection A549 cells were infected with NL09 and NL11 at (MOI = 0.1 or 3) and incubated for 72 h in presence of NRTKIs at [0.25x]_max_ (gray) and [0.5x]_max_ (white) concentrations. At 24, 48, and 72 hpi, supernatants were collected and viral titers quantified by TCID_50_/mL assay (n = 4). L.o.d.: limit of detection. Means ± SD are shown. ∗, p < 0.05; ∗∗, p < 0.01; ∗∗∗, p < 0.001; ∗∗∗∗, p < 0.0001. p values were determined by Mann-Whitney tests compared to untreated cells.

We excluded RX and TF from subsequent studies due to reduced viability and limited effects on viral titers. To determine if the NRTKI’s antiviral effects were MOI-dependent, we infected A549 cells with either a high MOI (MOI = 3) or a low MOI (MOI = 0.01) in the presence or absence of NRTKIs [0.5x]_max_. The effect on the seasonal H3N2 (NL11) strain was more pronounced compared to that on the pandemic H1N1 (NL09) strain; presumably due to the faster growth kinetics of NL11 compared to NL09 (Figure 2). However, NRTKI treatment of cells infected at high (3) or low (0.01) MOI resulted in viral titer reductions of ≥10-fold (1-log_10_) (Figure 2). Treatment with DF, BO, or NI had larger effects on early (24 hpi) viral replication, especially in NL11 infected cells. Although peak titers of untreated cells infected with NL11 were similar at either MOI, cells infected at MOI = 0.1 exhibited the greatest reduction (∼1,000-fold or 3-log_10_). AC, IB, and SA treatment had less of an impact on viral titers than DF, BO, or NI, but viral titer reductions were still up to 100-fold (2-log10) (Figure 2).

### Validation of NRTKIs as IAV antivirals in hPCLS

**Figure 3:**
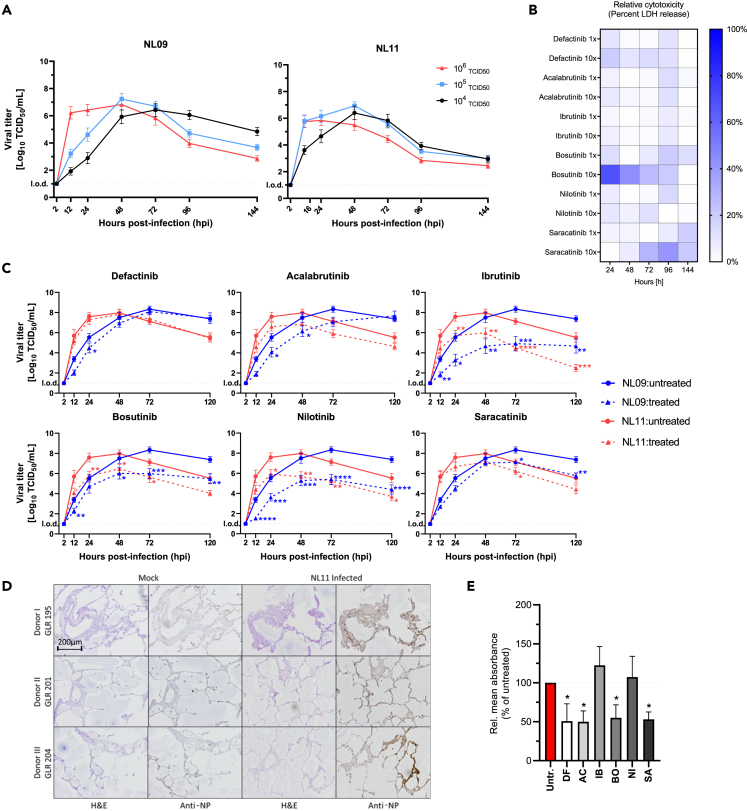
NRTKIs inhibit *ex vivo* IAV infection (A) hPCLS were infected with NL09 and NL11 (10^4^, 10^5^ or 10^6^ TCID_50_/200ul). Viral titers were quantified by TCID_50_/mL assay at 2, 16, 24, 48, 72, 96, and 144 hpi (8 donors; n = 24/virus). (B) Heatmap visualization of NRTKI cytotoxicity in hPCLS treated with [1x]_max_ and [10x]_max_ concentration up to 144 h. At each time point, LDH release was measured using LDH-Glo Cell Viability Assay and normalized to DMSO control and relative to 1% Triton X-100 treated cells (positive control) (8 donors/n = 24). (C) hPCLS were infected with NL09 or NL11 (10^5^ TCID_50_/200ul) and incubated for 120 h with NRTKIs (Defactinib 50uM; Acalabrutinib 5uM; Ibrutinib 5uM; Bosutinib 5uM; Nilotinib 10uM; Saracatinib 0.125uM). Virus was quantified by TCID_50_/mL assay at 2, 12, 24, 48, 72, and 120 hpi (3 donors; n = 6/condition). (D) NL11 infected hPCLS were fixed 120 hpi and PFA-fixed paraffin-embedded (PFPE) PCLS were cut into 2 μm thick section. H&E staining and viral anti-NP staining (brown) at 10× magnification (Scale bar: 200 um). (E) Semi-quantitative analysis of virus infection (anti-NP staining) was performed for tested NRTKIs and normalized to tissue-area in each section using FIJI image-analysis software. Means ± SEM are shown. l.o.d.: limit of detection. ∗, p < 0.05; ∗∗, p < 0.01; ∗∗∗, p < 0.001; ∗∗∗∗, p < 0.0001. Significance (p values) was determined by Mann-Whitney tests compared to untreated cells.

We utilized human precision-cut lung slices (hPCLS) as a biologically relevant *ex vivo* model that more faithfully represents lung tissues than either 2D monolayer or 3D air-liquid-interface (ALI) cultures.^33^^,^^34^ We used hPCLS cultured up to 4 weeks; no gross alterations in cell type or morphology were observed and cilial beating was observed. We infected hPCLS with 10^4^, 10^5^, or 10^6^ TCID_50_/200 μL of NL09 or NL11 to identify an infectious dose where peak titers of NL09 or NL11 are synchronized; these strains have different replication kinetics *in vitro*. To limit donor-heterogeneity effects, we used hPCLS from 8 donors (n = 24/virus). Highest peak titers were achieved at 48 hpi following infection with either 10^4^ or 10^5^ doses of NL11, but only in the 10^5^ dose of NL09 (Figure 3A); therefore, the 10^5^ dose was used in all subsequent hPCLS infections.

Next, we determined the tolerability of our hPCLS to either [1x or 10x]_max_ NRTKI concentrations by measuring lactate dehydrogenase (LDH) release into the culture supernatant as an indicator for cytoxicity. As a positive control for cytotoxicity, hPCLS were treated with 0.1% Triton X-100; DMSO-treated hPCLS were used as a vehicle control (Figure 3B). Our cytotoxicity cut-off was 20% of the positive control treatment; none of the NRTKIs surpassed this cut-off at [1x]_max_. However, 10x_max_ concentrations of DF (50 μM), BO (50 μM), and SA (1.25 μM) showed significantly higher cytotoxicity (>20%); we therefore, only used 1x concentrations of these NRTKIs in subsequent hPCLS experiments (Figure 3B).

Next, hPCLS from 3 donors (n = 6/virus/condition) were infected with 10^5^ TCID_50_ of NL09 or NL11 and treated with NRTKIs (DF 5 μM; AC 5 μM; IB 5 μM; BO 5 μM; NI 10 μM; SA 0.125 μM). All tested NRTKIs reduced viral titers by at least 10-fold or 1-log_10_ (DF treatment) to more than 1,000-fold or 3-log_10_ (IB and NI treatments) (Figure 3C). However, while this effect was significant throughout the infections with either NL09 or NL11 following IB, BO, and NI treatments, AC and DF treatment was only significant at 24 and 48 hpi. Moreover, unlike what we observed in A549 cells, IB-, BO-, and NI-mediated IAV inhibition was observed within 12 hpi and maintained at 120 hpi; which was after the times of peak titers (48–72 hpi) (Figure 3C).

Next, we assessed NRTKI effects on the viral spread and associated damage to the epithelium. At 120 hpi, mock- and IAV-infected hPCLS (n = 3/virus/condition) were fixed and paraffin-embedded. H&E staining did not suggest gross alterations in cell composition or epithelium in mock-infected cells indicating acceptable viability of hPCLS; typical morphological changes associated with IAV infections were observed. Only data for NL11 is shown as we did not observe significant differences in titer reductions between NL09 and NL11 (Figure 3D). We observed IAV-specific staining in all observed cell types including type I/II pneumocytes and endothelial cells in consecutive sections from those H&E stained. Additionally, staining intensity and quantity were reduced in NRTKI-treated hPCLS compared to untreated hPCLS (Figure 3D). Semi-quantitative analysis of acquired images indicated that DF, AC, BO, and SA treatment significantly reduced infectivity by >50%, whereas IB and NI treatment had limited effects on infectivity (Figure 3E).

### Stability of NRTKI inhibition

**Figure 4:**
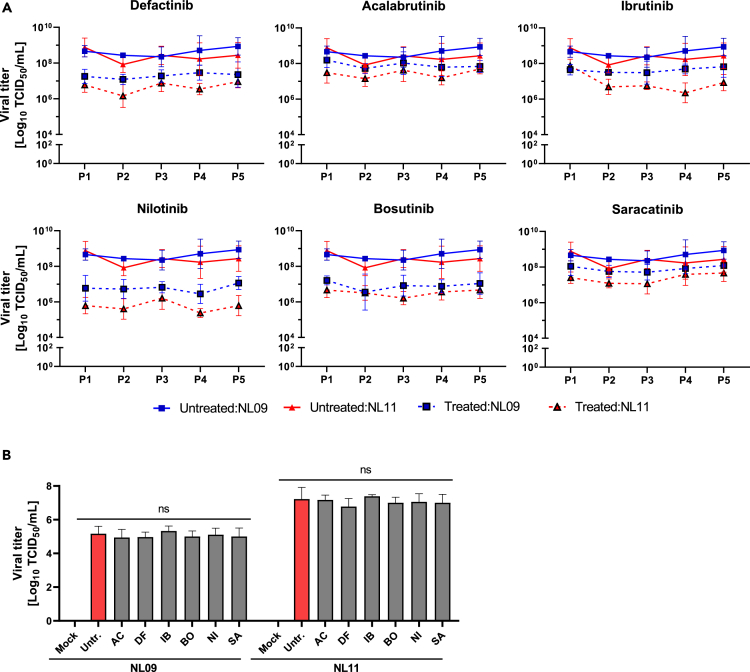
NRTKI inhibition of IAV is stable (A) Stability of SMKI treatment on NL09 and NL11 replication was determined by serial passaging (5 times) using MDCK cells infected at MOI = 0.001 for 72 h in the presence of the [1x_max_] NRTKI concentrations (n = 4) at each passage. At each passage, virus titers were quantified to inoculate the next passage at MOI = 0.001 again. Means ± SD are shown. (B) NL09 and NL11 virus stocks were pre-incubated with control (DMSO) or the [1x_max_] concentration of respective NRTKI for 4 h at 37°C and A549 cells were then infected using a 1:1000 dilution and incubated for 72 h, after which, virus titers were determined by TCID_50_/mL assay (n = 3). Means ± SD are shown. p values were determined by Welch t-tests compared to untreated cells; ns, not significant.

Host-directed antivirals/therapeutics likely have a higher barrier of resistance than their virus-targeted counterparts. To determine the stability of NRTKI antiviral effect, we phenotypically assessed the emergence of resistant escape variants.^35^^,^^36^ We passaged both NL09 and NL11 viruses (MOI = 0.001) in the presence of NRTKIs [1x]_max_ in MDCK cells for 5 passages. Untreated virus stocks were also passaged as a control. In untreated passages, virus titers for both NL09 and NL11 were similar from passages 1 to 5 and were significantly lower in all treated passages (Figure 4A); this reduction was comparable to that observed in A549 cells. Viral titers were stable at all passages indicating no resistance mutations were acquired. Next, we ruled out NRTKI virucidal activity or direct NRTKI-virus interactions that may inhibit attachment or entry. Pre-treatment of virus stocks with NRTKIs [1x]_max_ for 2 h prior to A549 infection had no effect on viral titers, indicating that the observed effects are due to host-cell effects (Figure 4B).

### Selected NRTKIs inhibit viral entry

**Figure 5:**
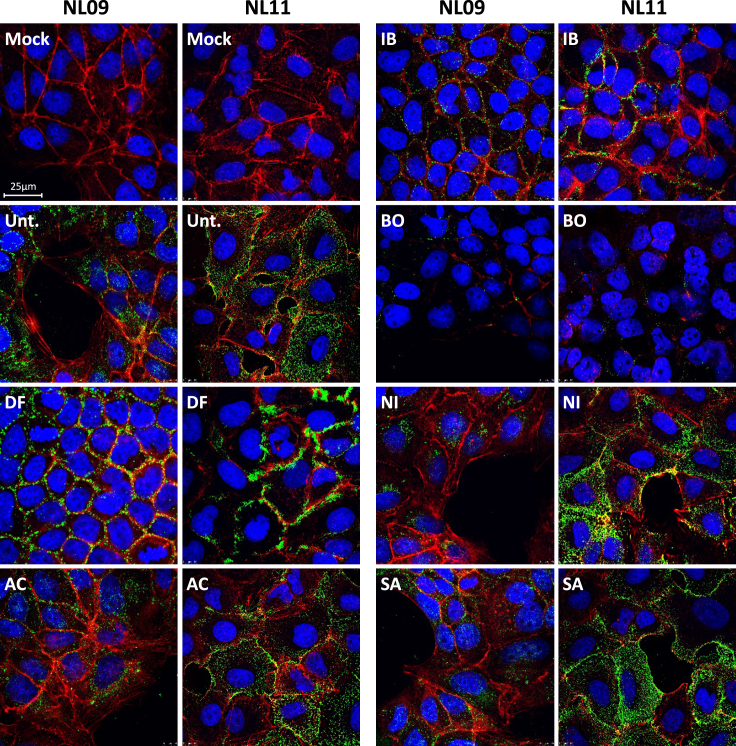
NRTKI-specific effects on viral entry A549 cells were pretreated with NRTKIs for 2 h then infected with NL09 or NL11 strains (MOI = 10) for 0.5 h +/− NRTKIs [1x_max_]. Cells were fixed and permeabilized and virions detected by anti-NP (green) antibody, F-Actin detected by ActinRed-555 (red), and nuclei detected using NucBlueLive ReadyProbes (blue). Virion localization was visualized by confocal microscopy (n = 2) (Scale bar: 25um).

Kinases regulate every step of the infection cycle and a single kinase can affect multiple steps.^28^^,^^29^ To determine the effect of our NRTKIs on viral entry, A549 cells were pretreated for 2 h, then infected with NL09 or NL11 (MOI = 10). At 0.5 hpi, cells were fixed, stained to detect viral NP, F-actin, and nuclei, and analyzed by confocal microscopy. We observed significant retention at the membrane and cell periphery following DF and IB treatment (Figure 5). Surprisingly, no virus was detected in response to BO treatment and the F-actin network was not detectable. Given the sustained viability of BO-treated cells, it is likely that the altered actin dynamics were tolerated. No significant changes were detectable in AC-, NI-, or SA-treated cells suggesting they did not affect viral entry (Figure 5).

### NRTKIs exert differential effects on IAV polymerase activity

**Figure 6:**
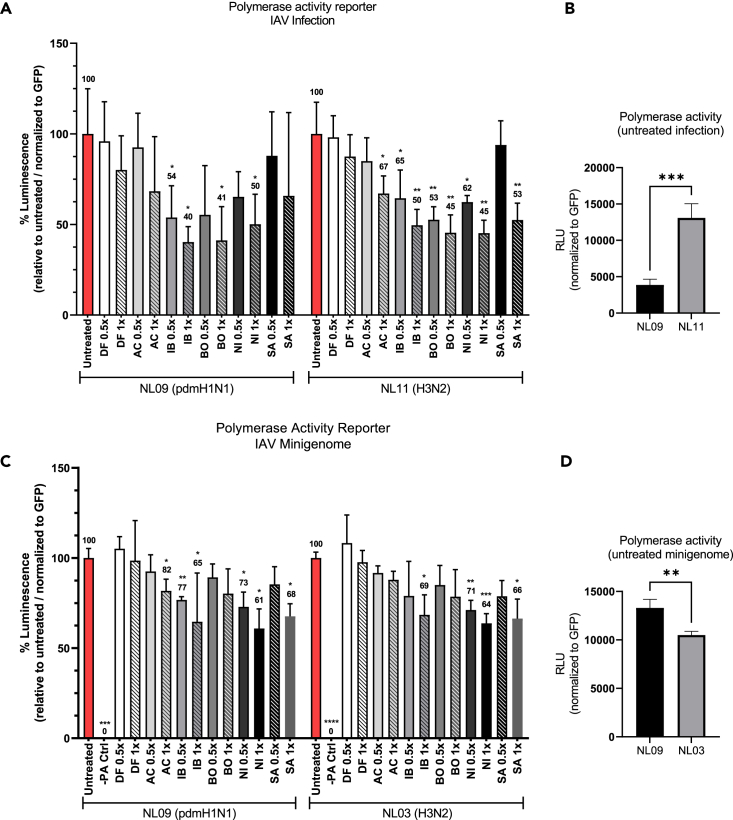
NRTKIs affect IAV RNA replication (A) A549 cells were transfected with pPOLI-358-FFluc and pmaxGFP plasmids. At 24 hpt, cells were infected with NL09 or NL11 at MOI = 1 +/− indicated NRTKIs at [0.5x or 1x]_max_ concentrations. At 48 hpt (24 hpi), luciferase activity was measured and normalized to GFP expression (MFI). (B) GFP-normalized polymerase activity of untreated NL09-or NL11-infected cells is shown. (C) A549 cells were transfected with pPOLI-358-FFluc and pmaxGFP plasmids and co-transfected with NL09 or NL03-minigenome plasmids. At 6 hpt, NRTKIs were added to the medium. At 30 hpt (24 h of treatment), luciferase activity was measured and normalized to GFP MFI. Bars indicate values relative to untreated cells normalized to GFP. (D) GFP-normalized polymerase activity of untreated NL09 or NL03 minigenome transfected cells is shown. Triplicate measurements from triplicate samples (n = 3); error bars indicate ±standard deviation (SD). ∗, p < 0.05; ∗∗, p < 0.01; ∗∗∗, p < 0.001; ∗∗∗∗, p < 0.0001. p-values determined by Brown-Forsythe and Welsh ANOVA compared to untreated.

We next assessed the effect of our NRTKIs on viral polymerase activity. The pPOLI-358-FFLuc reporter plasmid, which encodes a firefly luciferase gene under the control of the viral nucleoprotein (NP) promoter, and luciferase activity is a surrogate for viral polymerase activity.^37^^,^^38^^,^^39^ We first compared the effect of NRTKIs on polymerase activity during infection. We observed a significant reduction in polymerase reporter activity in response to AC (NL11 only), IB (NL09 only), BO, NI, and SA (NL11 only) (Figure 6A). Although the magnitude of reduction was higher in NL09-infected than NL11-infected cells, a significant reduction was more readily observed in NL11-infected cells at lower NRTKI concentrations. DF reduced reporter activity (NL09 = 20%, NL11 = 13%); however, this reduction was not statistically significant. At 24 hpi, a 3-fold increase in reporter activity could be observed in untreated NL11-infected cells over untreated NL09-infected cells; this is in line with faster replication kinetics of NL11 compared to NL09 (Figure 6B).

To better dissect the direct effect on viral polymerase activity in absence of NRTKI-effects on viral entry and host responses, we determined polymerase activity using an established minigenome system that only expresses the viral replication complex proteins (NP, PA, PB1, and PB2). We used the minigenome from the related H3N2 strain NL03 as we did not have access to the NL11 minigenome plasmids; both strains are known to exhibit similar replication kinetics. In this context, AC (NL09 only), IB, NI, and SA treatments significantly reduced polymerase activity (Figure 6C). In contrast to infected cells, the magnitude of reduction in polymerase activity was comparable in NL09- and NL03-minigenome transfected cells. Interestingly, polymerase activity was significantly higher in untreated H1N1 (NL09) than in H3N2 (NL03) minigenome-transfected cells (Figure 6D).

### NRTKIs do not affect innate immune responses during IAV infections

**Figure 7:**
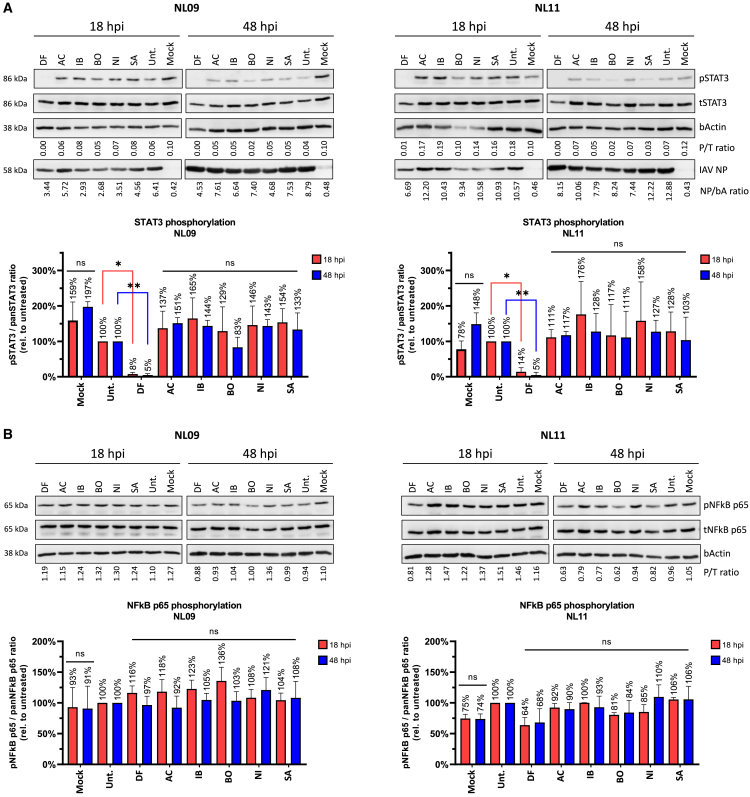
Effect of NRTKIs treatment on STAT3 and NFkB activation A549 cells were infected with NL09 or NL11 at MOI = 1, treated with NRTKIs at [1x_max_] concentration and total proteins isolated from whole cell lysate at 18 and 48 hpi. Immunoblot assay was performed for phospho/total STAT3, IAV-NP and bActin (A), and phospho/total NFkB, IAV-NP and bActin (B). *See also*Figure S3*.* All measurements were taken from two Western blots from two-independent experiments (n = 2). All values are relative to untreated virus-infected cells. P = phosphor, T = total. Error bars indicate ±standard deviation (SD). ∗, p < 0.05; ∗∗, p < 0.01; ∗∗∗, p < 0.001; ∗∗∗∗, p < 0.0001; ns, not significant (p > 0.05). p-values determined by Student’s *t* test compared to untreated virus-infected cells.

STAT3 is a regulator of inflammatory responses that is activated by phosphorylation of Y705 (STAT3pY705) to upregulate anti-apoptotic factors. This activation is less efficient in H1N1 infections than in H5N1 infections which delays apoptosis more efficiently. Therefore, we determined if NRTKI-mediated inhibition of viral entry and/or replication affected STAT3pY705. We assessed STAT3pY705 in A549 cells infected with either NL09 or NL11 (MOI = 1) in the presence or absence of NRTKIs at [1x]_max_. As expected, decreased STAT3pY705 was observed in untreated NL09-infected cells, and to a lesser degree in NL11-infected cells (Figure 7A), compared to mock-infected cells (18 h = 159%, 48 h = 197%). Surprisingly, only DF treatment resulted in a significant reduction of pSTAT3 relative to untreated infected cells (NL09 = 5%–8%, NL11 = 5%–14% of untreated).

NFkB Activation (NFkBpS536) serves opposing functions in early vs later times of infection and is therefore tightly regulated during IAV infection. We did not detect a significant increase in relative NFkBpS536 in NL09- or NL11-infected cells compared to mock-infected cells at 18 or 48 hpi following treatment with any of the NRTKIs (Figure 7B). We did observe a reduction in NFkB activation following DF treatment of NL11-infected cells (18 h = 64%, 48 h = 68% of untreated); however, this reduction was not statistically significant. We also confirmed that NFkB signaling is not impaired in our system as treatment with the synthetic dsRNA activator, poly(IC), induced NFkBpS536 in mock-, NL09-,and NL11-infected cells at 18 and 48 hpi (Figure S3).

## Discussion

Despite their clear susceptibility to rapidly arising resistance mutations, virus-targeted antivirals are still the only available class of antivirals against respiratory viruses like influenza. In this study, we screened FDA-approved NRTKIs currently in clinical use against cancers and autoimmune diseases for their antiviral potential against IAV infections. Six of eight tested NRTKIs showed potent *in vitro* inhibition of pandemic (H1N1) and seasonal (H3N2) IAV strains with little to no impact on cell viability *in vitro*. The robust potency was further validated and confirmed for five of six NRTKIs using a faithful *ex vivo* model of human PCLS. We identified the step(s) of the viral replication cycle affected by each compound. In doing so, we provide valuable information on the interplay of signaling pathways regulating these steps and the likely kinases involved.

We initially used A549 cells (ATII lung adenocarcinoma); however, due to potential biases associated with aberrant expression and kinase activity of cancerous cell lines, we validated NRTKI candidates using human PCLS (hPCLS) from 11 donors in total. Unlike 2D monolayers or 3D well-differentiated air-liquid interface (ALI) cultures, PCLS preserve native lung tissue architecture, cellular composition including endothelial, ATI and ATII epithelial cells, fibroblasts, and maintain native extracellular matrix.^33^^,^^34^^,^^40^ Moreover, tissue tropism and infectivity of certain viruses may not be accurately represented *in vitro* due to the absence of relevant cell-cell interactions that influence infectibility and host responses.^41^ Accordingly, we observed a similar discrepancy in which strain-dependent variances observed in NRTKI-treated A549 cells were not observed in hPCLS, suggesting that the differences between IAV strains in A549 might be an *in vitro* artifact. Moreover, we observed wide tissue tropism in hPCLS which suggests that while ATII cells may support more efficient infection, other cell types of the lung are readily infectible as well. Nevertheless, robust viral titer reductions in both systems following NRTKI treatment were observed that were not readily explained by reduction in infectivity or cell viability only. Defactinib (DF) < Acalabrutinib (AC) < Saracatinib (SA) < Bosutinib (BO) treatment had the smallest effect on viral titers, but each of these NRTKIs reduced hPCLS infectivity by ∼50%. Similarly, Ibrutinib (IB) and Nilotinib (NI) had the largest effect on viral titers and actually increased infectivity (NI:∼22%, IB = ∼7%). Together our data indicate that the reduction in infectivity of either A549 cells or hPCLS does not fully account for the potent reduction in viral titers. The rate at which viruses infect cells can indeed influence viral titers. However, the correlation between infectivity and titers is not absolute, and may only be readily observed when using a compound that targets the virus itself. Infectivity involves viral entry and replication at the very least, and in our assays, we carried out the infections at an MOI of 1 to allow for effects on virus spread. While the same number of cells may be infected, compound-specific effects on viral entry, polymerase activity, and/or virion assembly/egress could vastly differ and result in an uncoupling of the virus infectivity-titer correlation. Therefore, we do not believe that our findings regarding infectivity/viral titers are mutually exclusive or contradictory and support a *bona fide* effect of NRTKIs on viral entry and/or RNA replication.

Host kinases play a critical role in IAV entry, replication, and release as well as viral evasion/suppression of hosts’ immune responses; processes often requiring phosphorylation of viral proteins by mostly still unidentified kinases.^42^^,^^43^^,^^44^^,^^45^^,^^46^^,^^47^^,^^48^^,^^49^^,^^50^^,^^51^^,^^52^^,^^53^ Although there is a growing body of *in vitro* and *in vivo* evidence to support the therapeutic potential of kinase inhibitors, not a single SMKI has been approved or licensed for the treatment of influenza virus infection so far.^1^^,^^13^^,^^14^^,^^17^^,^^48^ SMKI selectivity remains a contentious topic and has been a hurdle to the pursuit of kinase inhibitors as antivirals. While compounds still in pre-clinical development phases require target validation, the selectivity of compounds in clinical use has been heavily investigated.^21^^,^^54^^,^^55^^,^^56^ Phosphorylation or activation of proteins/pathways beyond the intended target are often regarded as “off-target effects”. However, kinase-substrate interactions highlight extensive crosstalk between signaling pathways. Therefore, inhibition of an “off-target” kinase or pathway, should not be oversimplified and attributed to promiscuity of the SMKI in question; rather it is more likely evidence of an interaction between the intended target and the affected “off-target” signaling node. Two seminal studies collectively examined the selectivity of over 170 SMKIs against more than 440 kinases, covering ∼80% of the human kinome. Davis et al. compared inhibitor-kinase binding affinities, whereas Anastassiadis et al. used functional kinase inhibition assays. These studies suggest that while classes of SMKIs can inhibit multiple kinases within a single subfamily, inhibitors are selective against kinases outside that subfamily.^55^^,^^56^ Moreover, these studies likely overestimated “off-target” effects due to the use of truncated or fused recombinant proteins that may adopt altered conformations in the absence of regulatory domains (i.e., regulatory subunit of PI3K) or binding partners that affect substrate or ATP binding sites availability^21^; suggesting even greater selectivity than was proposed by those studies. Likewise, selectivity of clinically approved SMKIs was validated by super-resolution microscopy (dSTORM) to show the superior specificity and selectivity of fluorescently labeled SMKIs like gefitinib (EGFR inhibitor), over either fluorescent EGF ligand or EGFR monoclonal antibody.^57^ Therefore, SMKIs can be used as molecular beacons to probe host signaling pathways and delineate how they are regulated by kinases.

NRTKIs which target known effectors of actin reorganization and endocytosis have a significant effect on IAV entry. Indeed, we previously showed that targeting FAK using the pre-clinical inhibitor Y15, led to inhibition of PI3K-mediated endosomal trafficking of virions.^28^ Using the FDA-approved FAK inhibitor DF, we saw comparable effects on actin reorganization and viral entry as we previously observed using Y15.^28^ This is consistent with DF having the most effect at earlier time points in both A549 and hPCLS when reduction in viral entry may have a larger impact than at later time points. Indeed, we observed a reduction in infectivity in response to DF treatment which is consistent with the observed reduction of viral entry.

Cell-specific Bruton’s tyrosine kinase (BTK) isoforms are implicated in PI3K and PLCγ signaling that are either pro- or anti-apoptotic.^58^ IB and AC are high-affinity irreversible inhibitors of BTK; IB also inhibits EGFR activity.^59^^,^^60^ IB treatment reduces excessive neutrophil infiltration, acute lung injury, and subsequent ARDS; ultimately resulting in increased survival of mice severely infected with IAV.^27^ Given that IB inhibits both EGFR and BTK whereas AC selectively inhibits BTK, the IB-specific reduction in viral entry we observed, suggests this effect is mediated largely through inhibition of EGFR signaling. This is consistent with IAV-induced EGFR signaling which facilitates viral entry and activation of downstream pathways (Src, PI3K, and ERK) that promote efficient replication.^61^

BO, along with NI and SA, are second-generation Src inhibitors. BO also inhibits Abl kinase and to a lesser extent BTK. Growth factor RTKs like PDGFR and EGFR induce Src-mediated activation of PI3K/AKT, Ras-Raf-MEK-ERK, FAK, and STAT3.^62^ Src’s mostly proviral role during IAV infections is modulated by the viral NS1 protein.^13^^,^^63^ Abl inhibition by some avian IAVs results in significant pathology *in vitro* and *in vivo*; however, Abl’s role in human IAV infections is not fully understood.^64^^,^^65^ The direct interactions of a specific avian NS1 motif with Abl are necessary for the disruption of Abl kinase activity and result in significant cytopathic effect.^64^^,^^65^ Interestingly, this motif was present in the pandemic 1918 IAV strain when it first crossed into humans but was quickly lost during human adaptation so recent circulating human IAVs do not carry this motif suggesting that Abl kinase activity is important for human adaptation of IAVs. In the context of cellular functions, Abl is activated by both Src-dependent and -independent EGFR and PDGFR signaling. Among its functions is cytoskeletal reorganization which is at least partially mediated through Src signaling. Interestingly, Abl activity seems to positively regulate EGFR receptor endocytosis^66^; establishing a possible link for Abl to EGFR-mediated IAV entry. This is consistent with the significant reduction in viral titers we observed following BO treatment of hPCLS and A549 cells. BO treatment also resulted in a stark reduction in viral entry, concurrent to a complete absence of detectible actin filaments despite an increase in cell viability during infection. This is consistent with reports that show BO inhibition of Src activity can lead to altered actin dynamics or enhanced depolymerization of F-actin due to retention of alpha- and β-catenin at the cell membrane^67^^,^^68^; a process that may be influenced by Abl activity which is also affected by BO treatment.

We used a polymerase activity reporter and mini-genome systems to better dissected the effect of our NRTKIs on RNA replication and polymerase activity.^39^ Interestingly, in the context of viral infections, we detected higher polymerase reporter activity in NL11 (H3N2)-infected cells than in NL09 (H1N1)-infected cells. In contrast, the opposite was true when using the minigenome. Faster replication kinetics may be more susceptible to NRTKIs as a reduction in replication rate leads to exponential differences with time. This suggests that polymerase activity of NL09 is higher than NL11 and the faster kinetics in virus replication observed in NL11 infected cells may be due to more efficient virus entry, release, or immune evasion than NL09, and not polymerase activity. We previously demonstrated that FAK, in addition to its role in viral entry, also regulates *in vitro* polymerase activity of multiple IAV strains using the selective FAK inhibitor Y15 or dominant-negative kinase mutants.^29^ However, in contrast to our previous studies, we only observed a modest and non-significant effect on polymerase activity following DF treatment. O’Brien et al. showed that Y15 was a significantly more potent and selective inhibitor of FAK activity than DF which also targets the FAK-related kinase Pyk2.^69^ The disparity between a given SMKI’s binding affinity (K_d_) and its functional inhibitory concentrations can also be observed in the case of a single inhibitor targeting multiple kinases. For instance, the K_d_ of the multi-kinase inhibitor sunitinib for TrkC is 5.1 μM, but a 10-fold lower concentration (0.5 μM) is sufficient to inhibit >97% of its activity. In contrast, sunitinib’s K_d_ for PAK3 is 16 nM, but not even a 30-fold higher concentration (0.48 μM) has an effect on its activity.^54^ Therefore, the difference in potency of FAK inhibition by DF vs Y15 may account for the limited effect of DF treatment on IAV polymerase activity we observed.

Inhibition of Src by SA, BTK, and EGFR by IB, and BTK by AC had less of a significant effect on IAV polymerase activity indicating that the contribution of these kinases to host innate immune signaling does not directly affect IAV RNA replication. In contrast, inhibition of Abl and PDGFRα by NI treatment had the most significant reduction in IAV polymerase activity that was also strain independent. These data point to a role of PDGFRα in facilitating efficient IAV polymerase activity. This is consistent with previous findings that show inhibition of PDGFR by the RTK inhibitor A9, blocks RNA synthesis of all viral RNA species (vRNA, cRNA, and mRNA) independently of NFkB signaling.^44^ However, A9 also inhibits EGFR and is not selective for PDGFR isoforms; whereas NI is >25-fold more selective for PDGFRα than PDGFRβ.^70^ Several reports indicate that IAVs modulate antiviral NFkB activity to facilitate viral replication. Inhibition of NFkB results in reduced viral titers partly due to a disruption of vRNP nuclear export.^71^^,^^72^^,^^73^ Although we observed induction of NFkB activation by poly(IC) treatment in mock- and IAV-infected cells at 18 h but not 48 h, we did not observe a robust induction in NFkB phosphorylation in IAV-infected cells without poly(IC) treatment. This is consistent with published data and most likely due to the immuno-suppressive role of the viral NS1 protein.^74^

FAK also modulates cellular immune responses by regulating T cell-, B cell-, and macrophage-functions as well as RIG-I-Like antiviral signaling.^75^^,^^76^^,^^77^^,^^78^ We previously demonstrated FAK-dependent regulation of NFkB signaling and polymerase activity *in vitro* and NFkB-dependent proinflammatory responses *in vivo.* ^30^ In that study, FAK inhibition increased survival, reduced viral load and pathogenesis in a severe infection model. However, DF treatment did not affect NFkB phosphorylation in this study; likely due to the difference in FAK inhibition potency between Y15 and DF. Similarly, none of the other NRTKIs influenced NFkB activation suggesting that the mechanism by which these NRTKIs inhibit virus replication is independent of the NFkB-pathway. Considering the transient and biphasic nature of NFkB activation, we cannot rule out that strain-dependent differences in kinetics did not affect the magnitude or duration of NFkB activation we observed as has previously been described by others.^79^^,^^80^^,^^81^^,^^82^^,^^83^^,^^84^

STAT3 is an emerging regulator of IFN and inflammatory responses. A wide range of cytokine, growth factors, and RTKs activate STAT3 via JAK1/2/3 and Tyk2-dependent phosphorylation at Y705 (STAT3pY705).^85^ The role of STAT3 is not fully understood with opposing functions dependent on pathway partners; IL-6 mediated STAT3 activation is proinflammatory while IL-10 mediated STAT3 activation is anti-inflammatory.^85^^,^^86^ Although STAT3 is dispensable for IFN signaling, it is activated by IFN-I and serves as a negative regulator to fine-tune the IFN response (reviewed in^86^). Recent studies suggest that STAT3 activation is likely IAV subtype/strain-dependent as well as host/tissue-specific.^87^^,^^88^^,^^89^ Because STAT3 activation upregulates anti-apoptotic factors, H5N1-mediated STAT3pY705 allows prolonged viral production through a delay of apoptosis; H1N1 is less efficient at STAT3pY705 and triggers apoptosis earlier.^88^^,^^89^ Although the mechanism of differential suppression of STAT3 activation by IAV is not clear, it has been suggested to be mediated by NS1 ^74^. Interestingly, EGFR activation can result in Src/FAK/BTK mediated activation of STAT3, thereby modulating the IFN and proinflammatory responses. Moreover, EGFR/Src-mediated STAT3pY705 requires Pyk2 kinase activity, which can also mediate full STAT3 transcriptional activity via JNK, p38, or ERK activation.^90^ As expected of H1N1 and H3N2 infections,^88^^,^^89^ we observed limited levels of STAT3pY705 in untreated cells that were comparable to that observed following treatment with most NRTKIs. However, we observed significant suppression of STATpY705 following DF treatment (7–14% of untreated infected cells). This is consistent with the fact that DF inhibits both FAK and Pyk2 and suggests that IAV-induced STAT3pY705 requires FAK and/or Pyk2 activity.

In summary, we demonstrate that NRTKIs target kinases required for efficient IAV replication and represent promising drugs for the development of the next generation of antivirals. It is tempting to speculate on the molecular mechanisms and contribution of individual kinases to the antiviral effects observed following NRTKI treatment. The NRTKIs with the greatest effect had overlapping targets that mainly included EGFR, PDGFRα, and Abl. In contrast, NRTKIs that mainly target BTK or Src or the more distant FAK/PyK2 family had less robust, but still biologically significant, effects on viral replication. This data could be further used to fine-tune selectivity and potency for next-generation antiviral SMKIs and provide a rationale for combination therapy options to maximize SMKI antiviral efficacy. Surprisingly, our tested NRTKIs directly affected steps of the virus replication cycle with limited effects on proinflammatory host responses. Nicholas et al. (2015) used human cubic lung explants from young healthy donors to show the efficacy of a single pre-clinical vATPase inhibitor (TVB024) against IAV infection.^91^ Our study builds on and extends their findings by using precision-cut lung slices from 11 older donors to validate 6 FDA-approved NRTKIs. Given that most of our PCLS were obtained from lung cancer tumor resections, our donors tend to be older, are often smokers and suffer from either COPD or other respiratory pathologies. Although at first glance this may be perceived as a limitation of our model, we believe that these donors represent the “at risk” populations that would most benefit from IAV antivirals. Therefore, our data obtained from donor PCLS using these already FDA-approved NRTKIs as IAV antivirals are highly applicable to clinical settings. It should be noted however, that because these inhibitors target host factors, their therapeutic window is likely to be different from that of virus-targeted antivirals and pre/clinical studies must take this into account to establish efficacy.

In contrast to virus-directed IAV antivirals which are susceptible to resistance mutations, our tested NRTKIs data have a high genetic barrier for resistance based on their stability of IAV inhibition after 5 passages in the presence of each of our six NRTKIs. Although we cannot rule out NRTKIs-selected mutations, we did not detect a significant change in the magnitude of viral titer reductions across 5 passages, suggesting that no mutations conferring resistance accumulated in viruses passaged in the presence of SMKIs. Additionally, their established safety and bioavailability data further warrants clinical evaluation of these compounds as potential influenza treatments. Given that IAV infections are typically restricted to the respiratory tract, localized delivery of kinase inhibitors can limit potential cytotoxic effects. Finally, the local microenvironment must be considered to elicit balanced immune responses and avoid opposite or unintended consequences of promising SMKIs on resident and infiltrating immune cells. Because many viruses utilize the same (or related) host kinases to facilitate replication and transmission, our studies have broader implications for the potential use of these SMKIs to treat infections by other viruses.

### Limitations of study

Due to the dependence on available hPCLS, human lung samples of older (>58 years old) mainly male patients (10 male/1 female) undergoing tumor resection (8 tumor resections/3 IPF) were overrepresented. Also, A549 cells were originally derived from a 58 years old male patient with lung cancer. Although the female and the IPF patient samples showed no differences in their susceptibility or response to the NRTKI treatment, we cannot exclude potential bias of our data based on sex, medical condition, or age. Since older adults are at high risk for influenza, our findings are particularly relevant for this age group. IAV has multiple mechanisms to actively suppress p65 phosphorylation at specific times of infection. However, it is apparent that p65 phosphorylation in IAV-infected cells is not straightforward and a matter of debate. Differences in experimental conditions like strains used, multiplicities of infection and kinetics, as well as methods of measuring p65 activation (*e.g.*, p65 phosphorylation only, phosphor/total p65 ratio, p65 nuclear translocation), could be at the basis of the discrepancies between studies. Addressing these discrepancies is well beyond the scope of the current study.

## STAR★Methods

### Key resources table

**Table:** 

REAGENT or RESOURCE	SOURCE	IDENTIFIER
**Antibodies**		

Mouse monoclonal anti-IAV NP (Clone HB65)	ATCC	Cat#ATCC-HB-65
Rabbit polyclonal anti-IAV NP (Clone PA5)	ThermoScientific	Cat#PA5-32242; RRID:AB_2549715
Rabbit monoclonal anti-phospho-NF-κB p65 (Ser536) (Clone 93H1)	CellSignaling	Cat#3033; RRID: AB_331284
Rabbit monoclonal anti-phospho-Stat3 (Tyr705) (Clone D3A7) XP®	CellSignaling	Cat#9145; RRID:AB_2491009
Mouse monoclonal anti-NF-κB p65 (Clone L8F6)	CellSignaling	Cat#6956; RRID:AB_10828935
Mouse monoclonal anti-Stat3 (Clone 124H6)	CellSignaling	Cat##9139; RRID:AB_331757
Mouse monoclonal anti-bActin (Clone BA3R)	ThermoScientific	Cat#MA5-15739; RRID:AB_10979409
Goat polyclonal anti-Rabbit IgG (H + L) Cross-Adsorbed Secondary Antibody, Alexa Fluor 594	ThermoScientific	Cat#A-11005; RRID:AB_2534073
Goat polyclonal anti-Mouse IgG (H + L) Cross-Adsorbed Secondary Antibody, Alexa Fluor 488	ThermoScientific	Cat#A-11001; RRID:AB_2534069
Goat polyclonal anti-Mouse IgG (H + L) Cross-Adsorbed Secondary Antibody, HRP	ThermoScientific	Cat#G-21040; RRID:AB_2536527
Goat polyclonal anti-Rabbit IgG (H + L) Cross-Adsorbed Secondary Antibody, HRP	ThermoScientific	Cat#G-21234; RRID:AB_2536530

**Bacterial and virus strains**		

A/Netherlands/602/09	ErasmusMC	N/A
A/Netherlands/241/11	ErasmusMC	N/A

**Biological samples**		

hPCLS #183 (Age: 67yo/Sex: m/Condition: tumor)	Pathology MHH	GLR#183
hPCLS #184 (Age: 74yo/Sex: m/Condition: tumor)	Pathology MHH	GLR#184
hPCLS #189 (Age: 77yo/Sex: m/Condition: tumor) central	Pathology MHH	GLR#189C
hPCLS #189 (Age: 77yo/Sex: m/Condition: tumor) periphery	Pathology MHH	GLR#189P
hPCLS #189 (Age: 77yo/Sex: m/Condition: tumor) airway	Pathology MHH	GLR#189AW
hPCLS #191 (Age: 60yo/Sex: f/Condition: tumor)	Pathology MHH	GLR#191
hPCLS #195 (Age: 66yo/Sex: m/Condition: tumor)	Pathology MHH	GLR#195
hPCLS #766 (Age: 59yo/Sex: m/Condition: IPF) central	Pathology MHH	GLE#766C
hPCLS #766 (Age: 59yo/Sex: m/Condition: IPF) periphery	Pathology MHH	GLE#766P
hPCLS #766 (Age: 59yo/Sex: m/Condition: IPF) airways	Pathology MHH	GLE#766AW
hPCLS #205 (Age: 65yo/Sex: m/Condition: tumor)	Pathology MHH	GLR#205

**Chemicals, peptides, and recombinant proteins**		

Defactinib (VS-6063)	Selleckchem	Cat#S7654
Bosutinib (SKI-606)	Selleckchem	Cat#S1014
Nilotinib (AMN-107)	Selleckchem	Cat#S1033
Saracatinib (AZD0530)	Selleckchem	Cat#S1006
Acalabrutinib (ACP-196)	Selleckchem	Cat#S8116
Ibrutinib (PCI-32765)	Selleckchem	Cat#S2680
Ruxolitinib (INCB018424)	Selleckchem	Cat#S1378
Tofacitinib (CP-690550)	Selleckchem	Cat#S2789
NucBlue™ Live ReadyProbes™	ThermoScientific	Cat#R37605
ActinRed™ 555 ReadyProbes™	ThermoScientific	Cat#R37112

**Critical commercial assays**		

CellTiter-Glo® 2.0 Cell Viability Assay	Promega	Cat#G9241
LDH-Glo™ Cytotoxicity Assay	Promega	Cat#J2380
ONE-Glo™ Luciferase Assay	Promega	Cat#E6110
Pierce™ Detergent Compatible Bradford Assay Kit	ThermoScientific	Cat#23246

**Experimental models: Cell lines**		

Human: A549	ATCC	CCL-185
Dog: MDCK	ATCC	CCL-34

**Recombinant DNA**		

pMAX GFP	Lonza	Cat#V4XC-2012
pPOLI-358-FFLuc reporter plasmid	Azzeh et al., 2001^37^; Deng et al., 2006^38^; Hoffmann et al., 2008^39^	N/A
A/Netherlands/602/09 PB2	ErasmusMC	RF1239PB2
A/Netherlands/602/09 PB1	ErasmusMC	RF1240PB1
A/Netherlands/602/09 PA	ErasmusMC	RF1241PA
A/Netherlands/602/09 NP	ErasmusMC	RF1243NP
A/Netherlands/213/03 PB2	ErasmusMC	RF600PB2
A/Netherlands/213/03 PB1	ErasmusMC	RF601PB1
A/Netherlands/213/03 PA	ErasmusMC	RF602PA
A/Netherlands/213/03 NP	ErasmusMC	RF604NP

**Software and algorithms**		

ImageJ	Schneider et al., 2012^92^	https://imagej.nih.gov/ij/
Fiji image processing package for ImageJ	Schindelin et al., 2012^93^	https://imagej.net/software/fiji/
Prism 9.0	GraphPad	https://www.graphpad.com/support/faq/prism-900-release-notes/
Image Studio™	Li-Cor	https://www.licor.com/bio/image-studio/
cellSens Software	Olympus	https://www.olympus-lifescience.com/de/software/cellsens/
CellCounting Macro	Grishagin, 2015^94^	https://doi.org/10.1016/j.ab.2014.12.007

### Resource availability

#### Lead contact

Further information and requests for resources and reagents should be directed to and will be fulfilled by the lead contact, Husni Elbahesh (husni.elbahesh@gmail.com).

#### Materials availability

This study did not generate new unique reagents.

### Experimental model and subject details

#### Cell lines

A549 cells were derived from ardenocarcinomic lung tissue from a 58 year old Caucasian male. Madin-Darby canine kidney (MDCK) cells were derived from epithelial cells from the kidney tubule of an adult Cocker Spaniel dog. MDCK cells were cultured in Dulbecco’s modified Eagle medium (DMEM; Gibco) supplemented with 10% fetal bovine serum (FBS) (ThermoScientific), 100 IU/mL penicillin (Gibco), 100 μg/mL streptomycin (Gibco), 2 mM glutamine (Gibco), and 1% nonessential amino acids (NEAAs) (Gibco). A549 cells were cultured in F-12 K-Nut Nutrient Mix medium (Gibco) supplemented with 10% FBS, 100 IU/mL penicillin, 100 μg/mL streptomycin, and 2 mM Glutamax (Gibco). All cells were incubated at 37°C and 5% CO_2_.

#### *Ex vivo* hPCLS model

Use of our human PCLS for *ex vivo* studies was previously described.^95^ Briefly, hPCLS were generated from lung tissues obtained from patients undergoing surgical operations at Hannover Medical School. Tissues used for PCLS generation that were obtained from lung tumor resections were confirmed as tumor-free by an experienced pathologist. The freshly obtained lung tissues were processed into circular slices that were 300 microns thick and 8 mm in diameter as previously described.^34^ All donors provided informed consent as approved by the Hannover Medical School Ethics Committee (Ethics vote #8867_BO_K_2020). PCLS were maintained in DMEM/F12 medium (Gibco) supplemented with 2 mM of HEPES (Gibco), 1× GlutaMAX, 100 U/ml penicillin and 100 μg/mL streptomycin in a humidified 37°C and 5% CO_2_ incubator.

#### Viruses

The pandemic H1N1 strain A/Netherlands/602/09 (NL09) and seasonal strain H3N2 A/Netherlands/241/11 (NL11) influenza viruses were obtained from the Repository of the National Influenza Center at the Erasmus Medical Center in Rotterdam, the Netherlands, and were grown on MDCKs for 48 h at 37 °C. Virus stocks and culture supernatants were stored at −80°C until further use. Virus yields were titrated on MDCK cells by 50% tissue culture infectious dose (TCID_50_)/mL method as described by Reed and Muench.^96^

### Method details

#### Inhibitors

Small molecule kinase inhibitors (SMKI) were all purchased from Selleckchem (TX, USA). Inhibitors were diluted in DMSO to a stock concentration of 10 mM and stored at −20°C upon usage.

#### *In vitro* cytotoxicity assays

*In vitro* cytotoxicity of SMKIs on mock-infected A549 cells was determined using CellTiter-Glo 2.0 (CTG) Cell Viability Assay (Promega). Roughly 80% confluent A549 cells cultured in a 96-well were washed twice with phosphate-buffered saline containing Mg^2+^/Ca^2+^ (PBS+/+) (Gibco). The cell were replenished with infection medium (F12K (Gibco) containing 0.1% [vol/vol] bovine serum albumin [BSA] (Sigma) and 50 ng/μL TPCK-treated trypsin (Sigma)) supplemented with small-molecule kinase inhibitor (SMKI) dilution series. The cell were incubated at 37°C and 5% CO_2_ for 72 h. At 72h cell-viability was evaluated using CTG according to manufacturer protocols. We defined the [1x]_max_ as the highest concentration resulting in > 95% cell viability following treatment.

#### *Ex vivo* cytotoxicity assays

Cytotoxicity of SMKIs on mock-infected PCLS was determined using the LDH-Glo Cytotoxicity Assay (Promega). hPCLS were placed in 48-well plates (1 hPCLS/well) and washed twice with PBS+/+ (Gibco). hPCLS were replenished with prewarmed culture medium (DMEM/F12 medium (Gibco) supplemented with 2 mM of HEPES (Gibco), 1 × GlutaMAX (Gibco), 100 U/ml penicillin (Gibco) and 100 μg/mL streptomycin (Gibco)) supplemented with SMKI [1x]_max_ and [10x]_max_. Supernatants of SMKI-treated and untreated hPCLS were collected and completely replaced with fresh pre-warmed infection medium containing SMKIs at the indicated concentrations. LDH levels were evaluated according to manufacturer’s protocol and calculated relative to the positive control (treated with 1% triton-X 100 for 30 min at 37°C).

#### Virus infections

A549 cells were plated on the day prior to infection so they were 80–90% confluent on the day of infection. For infections, viruses were diluted in infection medium (F12K medium (Gibco) containing 0.1% [vol/vol] bovine serum albumin [BSA] (Sigma) and 50 ng/μL TPCK-treated trypsin (Sigma)). The cells were inoculated with the virus at the indicated multiplicity of infection (MOI) for 1h at 37°C. The cells were washed twice with PBS+/+ (Gibco) to remove unbound virus and incubated in infection medium at 37°C in the presence or absence of SMKIs at the indicated concentrations. Supernatants were collected at 0, 24, 48, 72 h post-infection (hpi), and viral titers were determined by TCID_50_ assay in MDCK cells.^96^ Prism 9.0 (GraphPad) Heatmap function was used for visualization. The assay’s lower limit of detection (LoD) is 10^1^ TCID_50_/mL, and its upper LoD is 10^9.5^ TCID_50_/mL.

#### Immunofluorescent staining and imaging

To visualize virus infection, infected cells were fixed with 4% paraformaldehyde (4% PFA/PBS) (Roth) for 30 min at room temperature (RT), permeabilized with 0.1% Triton X-100 for 15 min at RT, washed with PBS and blocked with heat inactivated 5% horse serum (Sigma) in PBS (PBS-HS) at RT for 1h. Cells were then incubated with mouse monoclonal antibodies to IAV nucleoprotein (clone HB65, ATCC) diluted in PBS-HS at 0.2 μg/mL overnight at 4°C under constant agitation. Cells were washed and incubated with AlexaFluor-594 conjugated goat anti-mouse IgG antibody (0.2 μg/mL; ThermoScientific) and NucBlue Live ReadyProbes Reagent (ThermoScientific) for 1h at RT under constant agitation. Cells were washed 3 times with PBS (Gibco), images were captured using a Leica DMi8 fluorescence microscope and quantitative analysis was performed using ImageJ Threshold, Watershed, and Particle Analyser.

A previously described ImageJ Toolbox counting macro,^94^ was used to quantify the number of nuclei and the number of separate infected cells by analyzing the RAW image data for each channel (n = 4). The nucleus count was used to define the total cell number per 0.6 mm^2^. The NP staining was used to define the number of infected cells per 0.6 mm^2^. The ratio of infected to total cells was used to calculate Relative Infectivity. The total number of cells based on nuclei detected relative to mock-infected cells treated with the respective NRTKI was used to determine Relative Viability. Prism 9.0 (GraphPad) Heatmap function was used for visualization.

#### Immunohistochemistry staining

Mock- and virus-Infected PCLS were inactivated by fixation in 4% PFA/PBS (Roth) and paraffin-embedded into blocks. Tissue sections (2 μm thick) were cut from the paraffin-embedded blocks and subjected to Hematoxylin & Eosin (HE) staining using standard protocols. Immunostaining for IAV antigen was done using an HRP-conjugated anti-IAV NP antibody. Histological analysis was performed by an experienced pathologist blinded to clinical data and experimental setup using a routine diagnostic light microscope (BX43, Olympus). Representative images were acquired with an Olympus CS50 camera using Olympus CellSens software (Olympus). Semi-quantitative analysis of IAV NP signal was performed for all tested NRTKIs using FIJI image-analysis software.

#### Polymerase activity assay

Semi-confluent (∼70–80%) A549 cells (8×10^4^ cells in 24-well plates) were transfected using Lipofectamine LTX with the pPOLI-358-FFLuc reporter plasmid, which encodes a firefly luciferase gene under control of the viral nucleoprotein (NP) promoter (kindly provided by Megan Shaw)^37^^,^^38^^,^^39^; the Lonza pmaxGFP™ expression vector, was used as a transfection control.

For minigenome polymerase activity, a mix of plasmids encoding the PB2, PB1, PA, and NP genes of A/Netherlands/602/09 (H1N1) or A/NL/213/03 (H3N2) IAVs in quantities of 0.35, 0.35, 0.35, and 0.5 μg, respectively, were co-transfected with the reporter and control plasmids. At 6h post-transfection (hpt), the indicated SMKIs were added at [1x]_max_ and [0.5x]_max_ concentrations (see Figure 1A/Table 1) and at 30 hpt (24h of treatment), luciferase reporter activity was detected using the One-Glo luciferase assay system (Promega). GFP mean fluorescence intensity (MFI) and luciferase luminescence were measured using a Tecan multi-mode plate reader.

To measure polymerase activity during IAV infection, cells were infected at an MOI of 1 with NL09 or NL11 at 24h post-transfection (hpt) of the pPOLI-358-FFLuc reporter and the GFP plasmids in the presence or absence of SMKIs at the indicated concentrations as described above. At 48 hpt (24 hpi), luciferase reporter activity was detected using the One-Glo luciferase assay system (Promega). GFP mean fluorescence intensity (MFI) and luciferase luminescence were measured using a Tecan multi-mode plate reader.

#### Viral entry assay and confocal microscopy

A549 cells were seeded on 12.5-mm coverslips in 24-well plates. On the day of infection, cells were washed 3 times with PBS+/+ (Gibco) and incubated in infection medium in the presence or absence of kinase inhibitors for 2h. The cells were chilled on ice for 15 min and inoculated with virus (MOI = 10) in the presence or absence of the indicated SMKI concentrations at 4°C and on ice for 30 min. To limit receptor activation due to continuous viral-receptor engagement/internalization following the 4°C adsorption and to gently warm up the cells, unbound/noninternalized virus was removed by washing the cells twice with RT PBS+/+ (Gibco). The cells were then incubated with prewarmed infection medium containing the respective SMKIs at 37°C for 30 min. Cells were then fixed in 4% PFA for 30 min, permeabilized with 0.1% Triton X-100 at RT for 15 min, washed in PBS (Gibco), and incubated overnight at 4°C in blocking buffer (PBS-HS). The cells were then incubated with anti-IAV NP antibody (clone HB65, ATCC) diluted in blocking buffer for 1h at RT, washed 3 times with PBS (Gibco), and incubated for 1h at RT with AlexaFluor488-conjugated goat anti-mouse IgG secondary antibody (0.2 μg/mL; ThermoScientific) diluted PBS-HS. Cell nuclei and F-Actin were stained with NucBlue Live ReadyProbes (ThermoScientific) and ActinRed-555 ReadyProbes Reagent (ThermoScientific), respectively. Coverslips were mounted with Prolong mounting medium (Invitrogen), and cell images were acquired with a Leica TSC SP5 laser-scanning confocal system mounted on an upright Leica DM6000 CFS using a 63× oil immersion objective. The images were merged and analyzed with Leica LAS software using identical imaging settings across all experiments.

#### NRTKI resistance analysis

To assess the resistance barrier for our NRTKIs, we passaged our viruses five times in the presence or absence of submaximal inhibitor concentrations ([0.5x]_max_; see Figure 1A/Table 1). The parental viruses were also passaged under the same culture conditions in parallel in the absence of NRTKIs. Semi-confluent MDCK cells (∼10^6^ cells/well in 6-well plates) were infected with the pandemic H1N1 strain A/Netherlands/602/09 (NL09) and seasonal strain H3N2 A/Netherlands/241/11 (NL11) at MOI 0.001. At each passage, the cultures were maintained in 3 mL MDCK infection media at 37°C for 72h, in the presence or absence of the [0.5x]_max_ (see Figure 1A/Table 1) of respective candidate NRTKIs. Supernatants were harvested, clarified by centrifugation at 500 x g for 5 min at 4°C, and stored at −80°C until titration by TCID_50_ assay on MDCK cells. For the subsequent passage, cells were infected by using virus from the previous passage at MOI = 0.001.

#### SDS-PAGE and immunoblotting

Proteins were isolated from whole cell lysates using the M-PER Mammalian Protein Extraction Reagent (ThermoScientific). Proteins were quantified by Pierce Detergent Compatible Bradford Assay (ThermoScientific), separated by 8% SDS-PAGE, transferred onto a PVDF membrane and blocked overnight in blocking solution (TBS pH7.6, 0.05% Tween 20, and 5% w/v of nonfat dry milk). Primary antibodies were diluted in blocking solution overnight at 4°C: phos-NFkB p65 (Ser536) (93H1) Rabbit mAb (1:1000) (Cell Signaling), phos-Stat3 (Tyr705) (D3A7) rabbit mAb (1:1000) (Cell Signaling), Influenza A virus NP Antibody (PA5-32242) rabbit pAb (1:20,000) (ThermoScientific). Beta-Actin (BA3R) mAb (1:5000) was used as a loading control. HRP-conjugated secondary anti-rabbit or anti-mouse antibodies (1:20,000) (ThermoScientific) were diluted in blocking solution for 1 h at room temperature. Proteins were detected by chemiluminescence using the Super-Signal West Pico Plus (ThermoScientific) and SuperSignal West Femto Maximum Sensitivity Substrate (ThermoScientific) using a Li-Cor C-DiGit scanner. Band density was quantified using ImageJ to determine Phospho/Total ratios after b-actin normalization. When necessary, the imaged membrane was subsequently stripped using a mild water-based stripping solution (1.5% glycine; 0.1% SDS; 1% Tween 20; pH 2.2) and restained for total proteins using NFkB p65 (L8F6) mouse mAb (1:1000) (Cell Signaling) or Stat3 (124H6) mouse mAb (1:1000) (Cell Signaling).

### Quantification and statistical analysis

Statistical analyses with GraphPad Prism 9.0 included multiple *t* test, Brown-Forsythe and Welsh’s ANOVA tests and Dunnett’s T3 test for multiple comparisons. Values are represented as means standard deviations (SD) or standard error of the mean (SEM), with a *p* value of 0.05 considered statistically significant (ns = p > 0.05; ∗ = p ≤ 0.05; ∗∗ = p ≤ 0.01; ∗∗∗ = p ≤ 0.001; ∗∗∗∗ = p ≤ 0.0001). The performed tests and given significances are provided in the figure legends.

## Data Availability

• All data has been included in main figures or supplementary information. All data reported in this paper will be shared by the lead contact upon reasonable request. • This paper does not report original code. • Any additional information required to reanalyze the data reported in this work paper is available from the lead contact upon request.
