# Supplementary material for: FDA-approved Abl/EGFR/PDGFR kinase inhibitors show potent efficacy against pandemic and seasonal influenza A virus infections of human lung explants
Source: iScience. 2023 Feb 28;26(4):106309. doi: 10.1016/j.isci.2023.106309 (PMC10034449; doi:10.1016/j.isci.2023.106309)
Supplement: Document S1. Figures S1–S3 [file mmc1.pdf]

iScience, Volume *26*

Supplemental information

FDA-approved Abl/EGFR/PDGFR kinase inhibitors show potent efficacy against pandemic and seasonal influenza A virus infections of human lung explants

Robert Meineke, Sonja Stelz, Maximilian Busch, Christopher Werlein, Mark Kühnel, Danny Jonigk, Guus F. Rimmelzwaan, and Husni Elbahesh

SUPPLEMENTARY FIGURES

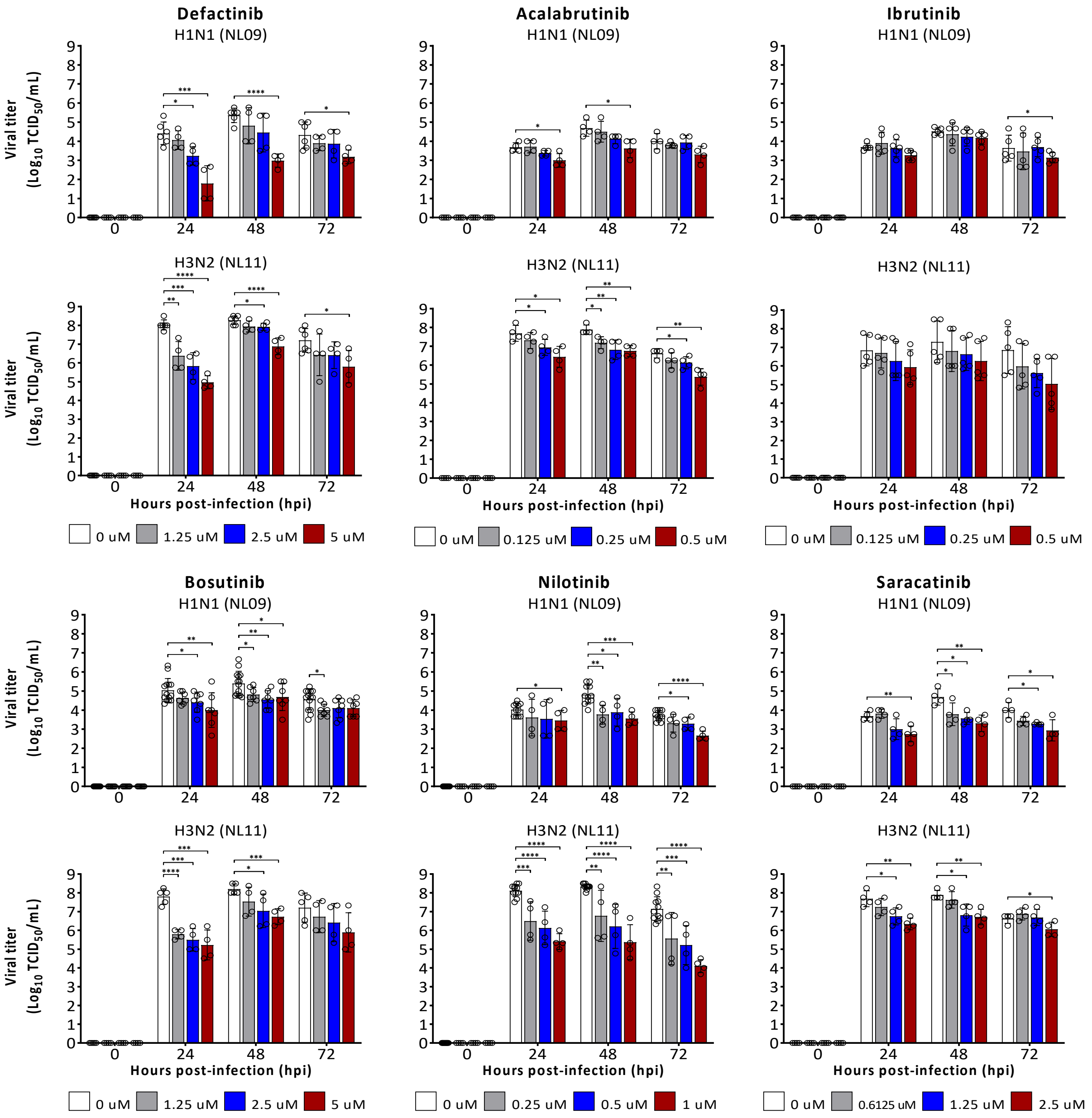

Figure S1: Effect of SMKI treatment on NL09 and NL11 replication at MOI=1, related to Figure 1. A549 cells were infected with NL09 or NL11 at MOI=1 and incubated for 72h +/- indicated NRTKIs at [0.25x, 0.5x or 1x]$_{max}$ concentrations. At 24, 48, and 72 hpi, supernatants were collected and viral titers quantified by $TCID_{50}$/ml assay (n = 4). Means ±SD are shown. *, P<0.05; **, P<0.01; ***, P<0.001; ****, P<0.0001. p-values were determined by Mann–Whitney tests compared to untreated cells.

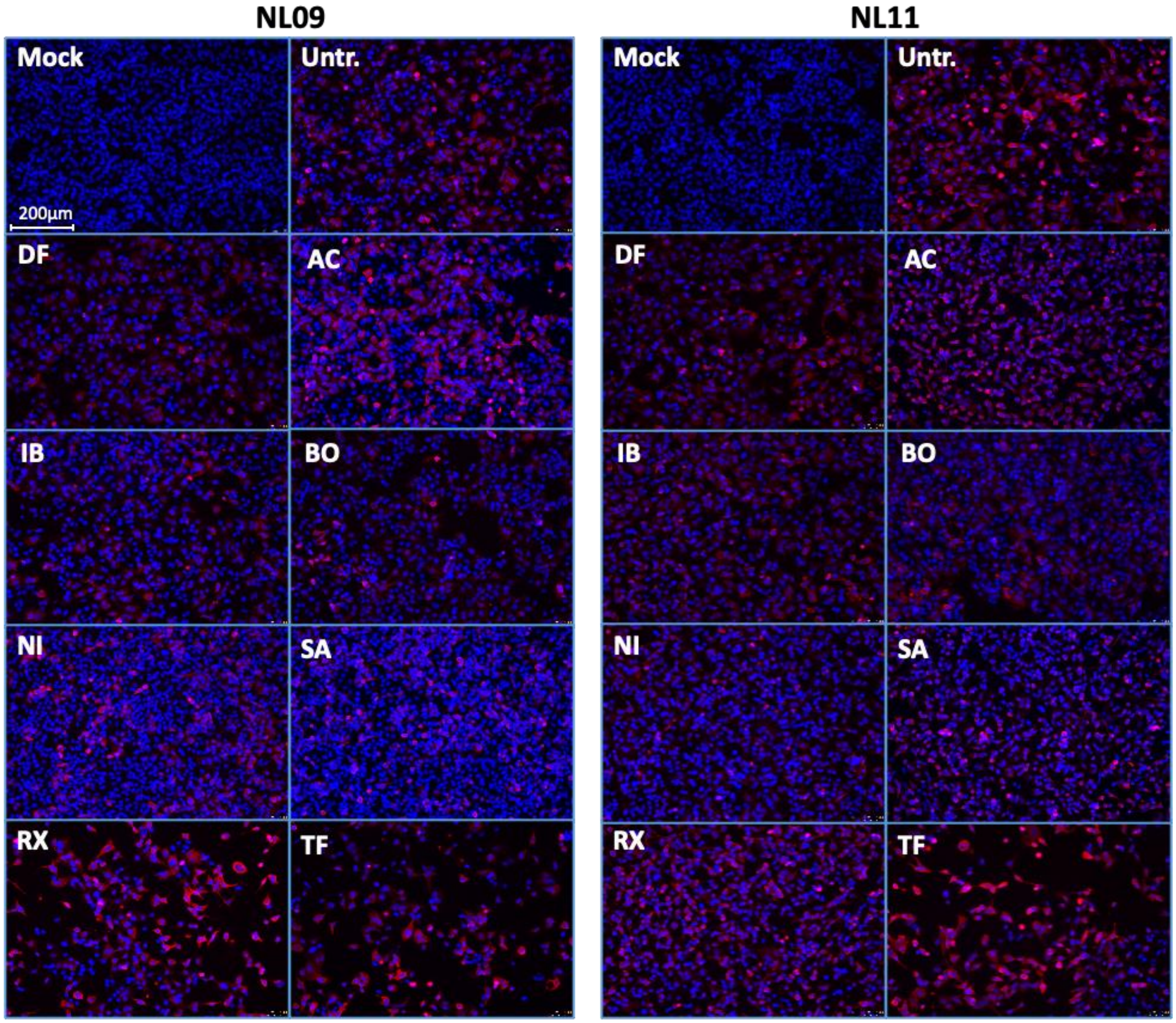

Figure S2. NRTKIs effects on cell viability and infectivity during infection, related to Figure 1. A549 cells were infected with NL09 or NL11 at MOI=1 +/- indicated NRTKIs at $[0.5x]_{max}$ concentration for 48h. Fluorescence microscopy pictures were captured using a Leica DMi8 fluorescence microscope (representative field shown from an n=4/condition). Virus-infected cells were detected by anti-IAV NP antibody (red), and nuclei were detected using NucBlue Live ReadyProbes (blue) (Scale bar: 200um).

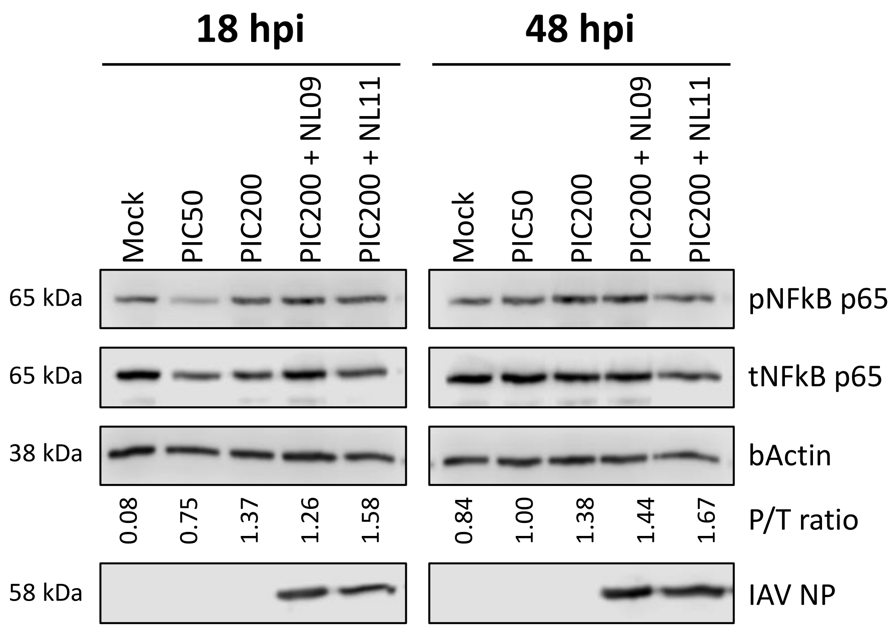

Figure S3. Effect of infection on NFkB activation in stimulated cells, related to Figure 7. A549 cells were infected with NL09 or NL11 at MOI=1 in the presence or absence of Poly(I:C). Total proteins were isolated from whole cell lysate at 18 and 48 hpi and immunoblot assay was performed for phospho- and total NFkB, IAV-NP and b-Actin. Chemiluminescence was detected using the Li-Cor C-DiGit. Band density was quantified using ImageJ to determine Phospho/Total (P/T) ratios after b-actin normalization. Blots are representative of two independent experiments (n=2). All values are relative to untreated cells. P=phosphor, T=total.
